# Clear lens phacoemulsification in the anterior lenticonus due to Alport Syndrome: two case reports

**DOI:** 10.1186/1752-1947-2-178

**Published:** 2008-05-27

**Authors:** Ghassem Amir Aslanzadeh, Davoud Gharabaghi, Niloofar Naderi

**Affiliations:** 1 Nikookari Eye Center, Tabriz University of Medical Sciences, Tabriz, Iran

## Abstract

**Introduction:**

Alport Syndrome has a prevalence of 1 case per 5,000 people and 85% of patients have the X-linked form, where affected males develop renal failure and usually have high-tone sensorineural deafness by age 20. The main abnormality is deficient synthesis of type IV collagen, the main component of basement membranes. Common ocular abnormalities of this syndrome consist of dot-and-fleck retinopathy, posterior polymorphous corneal dystrophy, and anterior lenticonus, but other ocular defects such as cataracts, posterior lenticonus, and retinal detachments have also been reported.

**Case presentation:**

We report two cases of anterior lenticonus due to Alport Syndrome and describe clear lens phacoemulsification and foldable intraocular lens implantation as an effective and safe refractive procedure in the four eyes of these two patients.

**Conclusion:**

All four eyes of the two patients were in good condition after surgery and achieved satisfactory optical and visual results and had no remarkable complications at six-months follow-up. Clear lens phacoemulsification with foldable intraocular lens implantation can be used as an efficient and safe procedure for vision disorders in these patients.

## Introduction

In 1927, Cecil A. Alport described three generations of a family with combinations of progressive hereditary nephritis and deafness and some ocular abnormalities. He noted that hematuria was the most common presenting symptom, and that men were affected more severely than women. Subsequently, many more families were described, and the eponym Alport Syndrome (AS) was coined in 1961 [1].

AS has a prevalence of 1 case per 5,000 people and 85% of patients have the X-linked form, where affected males develop renal failure and usually have high-tone sensorineural deafness by the age of 20 [2]. The main abnormality of this syndrome is deficient synthesis of type IV collagen, which is the main component of basement membranes in the human body. Common ocular abnormalities of this syndrome consist of dot-and-fleck retinopathy, posterior polymorphous corneal dystrophy, and anterior lenticonus (AL), but other ocular defects such as cataracts, posterior lenticonus, and retinal detachments have also been reported [1].

Homozygote males are usually severely affected but females tend to have a mild form, often with only microscopic hematuria and normal renal function. Common ocular abnormalities in the X-linked form are characteristically a dot-and-fleck retinopathy, less often AL, and rarely a posterior polymorphous corneal dystrophy [3].

AL is the pathognomonic feature of AS. This condition occurs in approximately 25% of X-linked AS patients. It is not present at birth, but appears later and worsens with age. It manifests by a slowly progressive deterioration of vision due to progressive myopia, requiring patients to change their glasses frequently. This condition is not accompanied by any eye pain, redness, night blindness, or color vision defects [1]. However, vision quality may be severely affected as a result of bulging of the anterior lens capsule and may require appropriate treatment including spectacle correction and/or surgery.

Herein, we report two cases of AL due to AS with severely impaired vision, and describe successful surgical treatment of these cases using clear lens phacoemulsification and foldable intraocular lens (IOL) implantation.

## Case presentations

Two brothers aged 24 and 26 years were referred to our eye hospital with bilateral visual acuity (VA) depression, not well corrected with glasses. The elder brother had a history of renal transplantation 4 years ago. His kidney pathology was not available at the time, but he had been told that his renal insufficiency was due to AS. He also had some hearing problems but was not using any hearing aids. The younger brother also suffered from renal disease and sonography revealed a kidney stone. He was using a hearing device for his serious bilateral hearing defect. They had one sister who had died due to renal failure. This seems unusual, because as mentioned before, the severe form of the condition is quite rare in women.

### Case 1: the older brother

**Figure 1:**
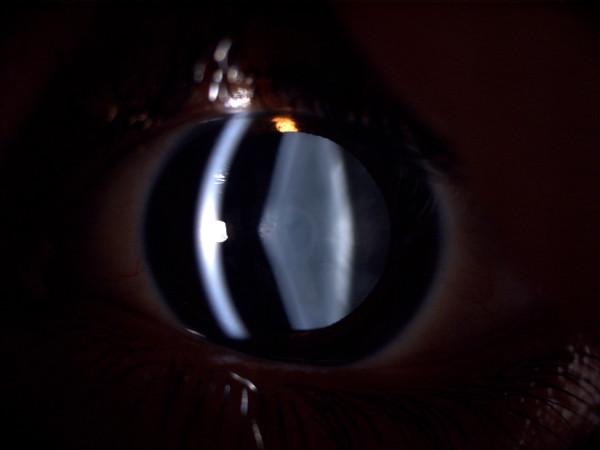
Slit lamp photo of the right eye of case 1.

**Figure 2:**
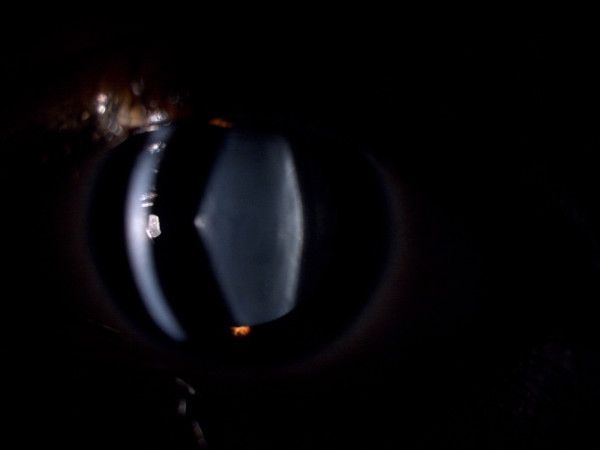
Slit lamp photo of the left eye of case 1.

Uncorrected visual acuity (UCVA) was determined by counting fingers (CF) 4 m for right, and 20/200 for the left eye. Best-corrected visual acuity (BCVA) was 20/200 for the right and 20/80 for the left eye. In slit lamp examination the AL was apparent in both eyes, but was more prominent in the right eye (Figures 1 and 2). Intraocular pressure (IOP) was normal.

The patient had no significant past ocular history other than he had worn spectacles for many years, but he had developed a myopic shift and some blurred vision quite recently. The ocular surface and corneas were normal. Oil droplet appearance was prominent in both eyes. Fundus examination revealed no specific pathology in the retina or optic disk. Clinically, our diagnosis was AL due to AS.

We planned a clear lens phacoemulsification with corneal incision and foldable IOL implantation (a refractive lens exchange) for his right eye. The procedure was performed under general anesthesia due to his hearing problem and fear of positive pressure. It was somewhat more time-consuming than ordinary cases. The forceps continuous curvilinear capsulorhexis (CCC) under viscoelastic material technique, hydrodissection, and phacoemulsification was carried out. The implant used was a hydrophobic acrylic foldable lens. There were no intraoperative complications other than a partial outgoing of capsulorhexis. Vision was 20/60 unaided (20/30 with pinhole) on the day after the operation. The postoperative period was uneventful. We continued the use of topical steroids four times a day for 3 weeks. We performed the same procedure on the left eye 4 weeks later. UCVA was 20/50 and improved to nearly 20/20 with a small astigmatic correction.

### Case 2: the younger brother

UCVA was 20/100 and 20/200 and BCVA was 20/30 and 20/40 for the right and left eye, respectively. In a slit lamp examination conjunctiva and cornea were normal, but protrusion of the lens capsule in both eyes was noticeable, consistent with the diagnosis of AL. Fundus and IOP were normal in both eyes. We performed the same procedure as in the first case, first on the left and then on the right eye, four weeks apart. Postoperative VA was 20/25 and 20/30 unaided in the right and left eye, respectively. Both eyes achieved a VA of 20/20 after a minor correction.

## Discussion

Lenticonus is bulging of the lens capsule and the underlying cortex. The diagnosis of lenticonus is essentially clinical, which is made by biomicroscopic examination. According to the localization of the conus, a distinction is made between anterior and posterior lenticonus. Whereas AL is a part of the AS, posterior lenticonus is not usually associated with a systemic disease [4].

Treatment of the visual problems in these patients is an important but secondary concern due to the seriousness of the systemic disease. However, double sensory loss (the vision and hearing defects) creates an urgent need for appropriate vision care [5].

John et al. [6] described bilateral phacoemulsification and implantation of foldable silicone intraocular lenses in a 25-year-old woman with AS and severe AL in 1995. They reported uncomplicated operations despite positive pressure, which they managed with viscoelastics, and the patient achieved excellent visual and refractive results.

AL has also been reported by other authors. Kato et al. [7] reported a case of AL, which was treated with phacoemulsification and IOL implantation. They advise caution while attempting intraocular lens implantation into the lens capsule of patients with AS, because the lens capsule may be fragile in these cases. The association of capsular fragility with anterior lenticonus due to AS has also been reported [8].

In our cases, we found that the anterior capsule was relatively elastic, making the capsulorhexis difficult technically. We started with a relatively smaller capsulorhexis and tried to pull it towards the center, and had a small peripheral widening of capsulorhexis, which was managed without serious complication. We used general anesthesia because of their hearing problem and to avoid the positive pressure that has been reported before [6]. We had no important intraoperative complications or significant posterior capsule opacification during a six-month period of follow-up after surgery.

Zare et al. [9] have also reported good results in 11 eyes of six patients with AS, which were treated by phacoemulsification. They recommend this procedure for AL patients.

## Conclusion

AL is a rare condition in which severe myopia and lenticular irregular astigmatism may decrease the patient's vision. It may be pathognomonic for AS. Correction of this type of visual disturbance is extremely difficult, but refractive lens exchange seems to be a valuable solution. The results of this study are in agreement with the previous reports [6-9]. In conclusion, we recommend clear lens phacoemulsification with foldable IOL implantation as a safe and efficient procedure in AL patients. We preferred to use general anesthesia for the procedure in our cases because of the hearing problems and other mentioned difficulties in our patients.

## Competing interests

The authors declare that they have no competing interests.

## Consent

Written informed consent was obtained from the patients for publication of these case reports and accompanying images. A copy of the written consent is available for review by the Editor-in-Chief of this journal.

## Authors' contributions

GAA performed the surgeries, participated in the follow-ups and drafted the manuscript, DG participated in the surgeries and reviewed the manuscript, NN participated in the surgeries and follow-ups, collected data, and provided the photos.
